# Structured data vs. unstructured data in machine learning prediction models for suicidal behaviors: A systematic review and meta-analysis

**DOI:** 10.3389/fdgth.2022.945006

**Published:** 2022-08-02

**Authors:** Danielle Hopkins, Debra J. Rickwood, David J. Hallford, Clare Watsford

**Affiliations:** ^1^Faculty of Health, University of Canberra, Canberra, ACT, Australia; ^2^Faculty of Health, Deakin University, Melbourne, VIC, Australia

**Keywords:** suicide prediction, suicide prevention, systematic review, structured data, unstructured data, meta-analysis

## Abstract

Suicide remains a leading cause of preventable death worldwide, despite advances in research and decreases in mental health stigma through government health campaigns. Machine learning (ML), a type of artificial intelligence (AI), is the use of algorithms to simulate and imitate human cognition. Given the lack of improvement in clinician-based suicide prediction over time, advancements in technology have allowed for novel approaches to predicting suicide risk. This systematic review and meta-analysis aimed to synthesize current research regarding data sources in ML prediction of suicide risk, incorporating and comparing outcomes between structured data (human interpretable such as psychometric instruments) and unstructured data (only machine interpretable such as electronic health records). Online databases and gray literature were searched for studies relating to ML and suicide risk prediction. There were 31 eligible studies. The outcome for all studies combined was AUC = 0.860, structured data showed AUC = 0.873, and unstructured data was calculated at AUC = 0.866. There was substantial heterogeneity between the studies, the sources of which were unable to be defined. The studies showed good accuracy levels in the prediction of suicide risk behavior overall. Structured data and unstructured data also showed similar outcome accuracy according to meta-analysis, despite different volumes and types of input data.

## Introduction

Globally, one person dies by suicide every 40 seconds, accounting for 800,000 preventable deaths per year worldwide (1). Despite increased awareness of suicide as a major cause of preventable death, responsive clinical training, reduction in mental health stigma, targeted research, and refinement of clinical psychometrics, it remains difficult to accurately and uniformly predict suicidal behavior (2).

The most common methods for assessing suicidal behaviors have traditionally been through clinical judgement and the use of clinical psychometrics, which are both dependent on client self-report and have been suggested to be of limited accuracy (2). Indeed, recent research concluded clinical judgment is no better than a chance at predicting suicide risk (3). One factor is that risk assessments require some cause for concern prior to their undertaking (i.e., some notion of the potential risk to prompt the clinician to use the psychometric). While other human factors that introduce error include time pressure and lack of clinician training and knowledge (4). In addition, even in organizations where the use of risk psychometrics is a standardized procedure during clinical sessions, other impediments exist including client intent to take their own life without interference (5).

The additional use of structured data (human interpretable such as risk psychometrics) offers increased risk accuracy, but these too are reliant on self-report. Structured psychometrics such as the Beck Depression Inventory (BDI) (6), the Suicide Risk Screener (SRS) (7), or the Patient Health Questionnaire (PHQ-9) (8), increase predictive power moderately, depending on the length of time to suicidal event (9–11). The increase in accuracy is likely due to the standardized and targeted approach of such risk psychometrics. Nevertheless, a groundbreaking study conducted by Pokorny (12) focused on the prediction of suicide in a veterans' population and classified 4,800 males as low or high risk of suicide using clinical psychometrics. At a five-year follow-up, Pokorny noted a high rate of false negatives in the low-risk group and that only a very small number of the high-risk group actually took their own lives, so there was a large rate of false positives in the high-risk group. This finding has been replicated repeatedly over the last few decades (13). As such, suicide risk predictions through psychometrics, as well as by clinical judgement, rarely accurately identify those individuals who go on to complete suicide, suggesting that different methods of assessment and complimentary ways to classify risk be investigated.

Although psychometric use increases the accuracy of suicide risk detection moderately over clinical judgement alone, research suggested this became redundant the closer an individual is to a suicidal event (10). This reduction in accuracy as suicidal behavior is proximal may be attributable to client help-negation, or a sense of hopelessness about intervention (5). Consequently, when an individual would be considered at the highest risk due to imminence, the accuracy of psychometrics has been suggested to reduce to around 50% (14).

The reasons for the limited ability to forecast suicide risk over time were revealed by a recent meta-analysis, which concluded that traditional risk factors were poor predictors (2). This may be because interactions between risk factors are complex and multi-directional. Franklin et al., (2) suggested that such complexity cannot be captured entirely through psychometrics or traditional statistical models, and proposed an investigation into newer, technology-based methods of attempting to predict suicidal behaviors. Perhaps clinical judgment and psychometrics have had impaired accuracy because the approach to prediction has been linear and limited by the capacity of human cognition. To combat these limitations, technological advances have begun to be applied to mental health, psychotherapy and, more recently, suicide prediction and management (15). Research into technological methods of predicting suicidal behaviors, which are not solely dependent on potentially inconsistent client reports, the limits of human cognitive processing or clinical judgement, could complement current methods (16).

Machine learning (ML) is a subfield of artificial intelligence (AI) and refers to the ability of computers to “learn” through algorithms (a defined set of instructions) on datasets (17, 18). There are two methods of ML, namely, the first uses labeled data for algorithms to learn to predict output from input (supervised learning), and the second uses unlabeled data where algorithms need to learn the structure from the input data to create and organize output data (unsupervised learning) (19). There are different types of algorithms that can be used in prediction, although investigation of algorithm type was not the focus of this review. Nevertheless, a recent paper by Jacobucci et al. (20) outlined potential inflated accuracy rates when certain types of algorithms were paired with optimism bootstrapping (a validation method). For consistency, papers were scanned for these pairings of algorithm/validation methods and three such papers were removed to minimize bias.

Algorithm outcomes are commonly measured by the area under the receiving operator characteristics curve (AUROC, or AUC) (21). A confusion matrix, which informs accuracy outcomes is arrived at in classification studies through a model's performance in classifying true positive, false positive, true negative, and false negative outcomes in a dataset. Use of AUC as an overall performance metric can be seen in psychology and other fields, such as medicine, to evaluate the accuracy of diagnostic tests and to differentiate case subjects from control subjects (3, 22). The higher the AUC, the better a model is at predicting an outcome, such as suicidal behavior. The AUC ranges from 0 to 1, in which <0.5 is below chance, >0.5 is considered to be chance level, >0.6 is considered poor, >0.7 is considered fair, >0.8 is considered good, and >0.9 represents excellent predictive ability (22).

There is a growing body of research on the accuracy of ML in suicide risk prediction conducted over the past 6 years. Previous studies have sought to develop algorithms or “models” in certain contexts (inpatient/outpatient, different countries, and with various populations); a few have attempted to validate their findings through repeated studies (23, 24); and several comprehensive systematic reviews have assessed accuracy of suicide risk prediction in a more generalized way (25, 26).

The aim of this review was to investigate the importance of data type on accuracy outcomes between structured data and unstructured data. Structured data can be defined as data that is simple enough for human understanding both in volume and structure (27). For the purpose of this review, we define structured data as purposeful, self-report, suicide risk, or psychometric instrument data completed by participants and used by algorithms to predict suicide attempts or death. Structured data is often obtained individually through clinical practice or research and is considered targeted data given the specific focus on an outcome, such as suicide risk. Conversely, unstructured data is defined as large volumes of information, from much bigger populations. Such data is comprised of all the information held by a specific service or database of an individual's health interactions over a period of time and can include the number of visits, medication prescriptions, unstructured clinical notes, demographic information, physical health data, and hospital records. Although unstructured data may also contain some structured data such as psychometric information, such is only a small part of clinical records within these studies and unstructured data is largely unorganized. Unstructured data are commonly comprised in Electronic Health Records (EHR's) and large population surveys. Therefore, structured data is targeted and specific to suicide risk through the use of more easily interpretable standardized psychometric questionnaires, whilst unstructured data contains potentially less targeted information, suggesting a point of comparison between these two groups.

To date, research comparing these different data sources has not been considered or synthesized. The current systematic review and meta-analysis address this gap, forming the main aim of this study, by reviewing, comparing, and integrating results of studies using the data categories of structured data and unstructured data, to compare outcomes for suicide risk prediction. Consideration of potential moderator variables on the accuracy of suicide risk prediction algorithms is also explored across the data sources as a secondary aim, given the potential for between study variance. Such variance (or heterogeneity) can be attributable to various causes such as demographic factors, study characteristics/design, chance, research environment, or prevalence (28). Therefore, analysis of traditional demographic suicide risk variables, such as sex and age (2), as well as study-specific variables, including study outcome (attempt/suicide behavior/death) and service location (inpatient/outpatient), and data type (structured and unstructured) are investigated.

This article comprises a description of the process of the selection of papers including the search strategy, inclusion and exclusion criteria, data extraction, and statistical analysis which are covered in the method section of the article. The outcomes are then presented in table form and within the body of the results section, with a focus on meta-analysis and meta-regression. Lastly, a discussion is presented based on the results, highlighting significant findings, strengths, and limitations of the review, areas of future research, and a conclusion.

## Method

A protocol for this systematic review was registered with PROSPERO (Registration Number CRD42020202768, dated 8 September 2020).

### Search strategy

A search of the following electronic databases was conducted to find relevant studies: CINAHL Plus with Full Text, MEDLINE, Computers and Applied Science Complete, Psych Articles, PsychINFO, and Psychology and Behavioral Sciences Collection. The search was conducted in March 2022 and was restricted to English-language, peer-reviewed articles published from 1 January 2000 to March 2022. The following subject terms and Boolean operators were used: (artificial intelligence^*^ OR machine learning^*^ OR ai OR a.i.^*^ OR m.l. OR ml) AND (“suicide risk” OR “suicide prediction” OR “suicide”*)*. A gray literature search was also conducted via Google Scholar. Keywords were selected given the type of paper and statistical analysis used, systematic review, and meta-analysis, whilst structured and unstructured were selected related to the data types used to differentiate between the two sources of data. The terms suicide prediction and suicide prevention were used in most included studies, highlighting these words as outcomes across included papers.

### Inclusion and exclusion criteria

Only quantitative studies were included. Studies that used any type of ML algorithm and different time points, but that could be categorized into either structured data or unstructured data, which produced confusion matrix figures and an overall accuracy outcome were included. Structured data was categorized as the use of one or more psychometric instruments to predict suicide risk, whilst unstructured data were those that used electronic health records, databases, or other large datasets. Critical analysis of each data type and data sources of each study are outlined in Table 1. Studies included in the review were those that predicted suicidal behavior—suicide attempt/risk behavior or death by suicide. Studies had to contain adequate numerical data. Those that did not contain adequate data and where authors did not respond to data requests were excluded.

**Table 1:** Critical analysis and data sources for structured and unstructured data types.

**Structured Data**	**Unstructured Data**
**Advantages**	**Advantages**
• Can be interpreted by humans in both volume and format, useful for individual level interactions. • Data is more precise and structured as focused on a specific topic. • Human to human interaction, which may improve accuracy of information as psychometric use can be guided by clinicians. • Standardized psychometric assessments can be used by different populations and are more generalisable.	• Data is a mixture of structured, semi-structured or unstructured and does not have to be organized. • Data is often found in large record systems that have been collected over time. • Human to computer interaction, analysis can be done without client involvement. • Data collection is fast and can be drawn from large survey and registry data. • May highlight risk for those who deny suicide thoughts and behaviors.
**Disadvantages**	**Disadvantages**
• Data collection is slower given it is purposefully collected, often on an individual basis. • Questions are obvious as to their intention, and outcomes may be easier to help-negate by denying suicide risk.	• Data are complex and of large volumes that rely on a machine to be interpretable. • Analysis of data must be standardized in different areas to be generalizable to different populations. • Machine Learning can be complicated to undertake and specific knowledge is required, restricting the useability.
**Structured Data Studies**		**Unstructured Data Studies**	
Barros et al., (29)	Outcome Questionnaire (OQ) State/Trait Anger Expression Inventory (STAXI-2) Reasons for Living (RFL) Depressive Experience Questionnaire Family APGAR	Barak- Corren et al., (30)	Partners Healthcare Research Patient Data Registry
Barros et al., (31)	Author developed 25-item risk instrument	Barak-Corren et al., (32)	Assessable Research Commons for Health Network
Burke et al., (33)	The Behavioral Health Screen (BHS)	Carson et al., (34)	Electronic Health Records Psychiatric Inpatient Unit
Delgado-Gomez et al., (35)	Personality and Life Events (PLE) Barratt Impulsivity Scale Social Readjustment Rating Scale Brown and Goodwin Scale of Aggression International Personality Disorder Examination Screening Questionnaire (IPDE-SQ)	Chen et al., (36)	Swedish National Registry Data
Hill et al., (37)	National Longitudinal Study of Adolescent to Adult Health	Cho et al., (38)	National Medical Check-up Data
Horvarth., (39)	Borderline Personality Diagnostic Data	Kessler et al., (40)	Historical Administrative Data System (HADS)
Jung et al., (41)	Korea Youth Risk Behavior Web-Based Survey (KYRBWS)	Kessler et al., (42)	Veterans' Health Administration System
Kim et al., (43)	The Minnesota Multiphasic Personality Inventory (MMPS-2)	Metzger et al., (44)	Electronic Health Records (EHR) Emergency Department Hospital
Morales et al., (45)	Outcome Questionnaire (OQ) State/Trait Anger Expression Inventory (STAXI-2) Reasons for Living (RFL) Depressive Experience Questionnaire Family APGAR	Sanderson et al., (46)	Five Administrative Health Care Systems
Naghavi et al., (47)	PTSD Checklist (PCL 5) Post-Traumatic Growth Inventory (PTGI) Patient Health Questionnaire (PHQ-9) Multidimensional Scale of Perceived Social Support (MSPSS) Positive Mental Health Scale (PMH) Suicide Behaviors Questionnaire- Revised (SBQ-R)	Sanderson et al., (48)	Five Administrative Health Care Systems
Oh et al., (49)	Emotional Regulation Questionnaire (ERQ) Academic Resilience Scale (ARS) Satisfaction with Life Scale (SWLS) Spontaneity Assessment Inventory (SAI) Anxiety Sensitivity Index (ASI) Subjective Happiness Scale (SHS) Social Support Inventory (SSI) Revised Life Orientation Test (LOT-R) Symptom Checklist Revised (SCL) Behavioral Inhibition Scale (BIS) Psychological Wellbeing Scale (PWS) Conner Davidson-Resilience Scales Positive and Negative Affect Schedule (PANAS) FACIT Purpose In Life (PIL) Cognitive Emotion Regulation Questionnaire (CERQ) Short Depression Happiness Scale (SDHS) Inventory of Interpersonal Problems (IIP) Childhood Trauma Questionnaire (CTQ) Life Events Checklist (LEC) Beck Depression Inventory (BDI) Functional Social Support Questionnaire (FSSQ) Body Appreciation Scale (BAS) Gratitude Questionnaire (GQ-6) Rumination Response Scale (RRS) Perceived Stress Scale (PSS) Test Anxiety Inventory (TAI)	Simon et al., (23)	Seven Health Record Systems
Passos et al., (9)	Structured Clinical Interview (SCID-I) Hamilton Depression Rating Scale (HDRS) Youth Mania Rating Scale (YMRS) Hamilton Anxiety Rating Scale (HARS)	Su et al., (50)	Connecticut Children's Medical Center Health Records
Rozek et al., (51)	Suicide Attempt Self-Injury Interview (SASII) Beck Scale Suicidal Ideation (BSSI-W) Insomnia Severity Scale (ISI) Beck Anxiety Inventory (BAI) Interpersonal Needs Questionnaire (INQ) Life Events Checklist (LEC) Beck Hopelessness Scale (BHS) Beck Depression Inventory (BDI) Post-Traumatic Stress Disorder Checklist (PCL-5) Alcohol Use Disorders Identification Test (AUDIT-C) Suicide Cognitions Scale (SCS)	Tsui et al., (52)	University Pittsburgh Medical Center Medical Archival System
Ryu et al., (53)	Survey question about suicide attempts.	Van Mens et al., (4)	Nivel Primary Care Database
Shen et al., (54)	Self-Rating Anxiety Scale (SAS) Self-Rating Depression Scale (SRD) Epworth Sleepiness Scale (ESS) Adult ADHD Self-Report Scale Symptoms Checklist (ASRS) Self-Esteem Scale (SES) Conner Davidson Resilience Scale (CD-RISC)	Van Mens et al., (4)	Scottish Wellbeing Study
		Zheng et al., (55)	Berkshire Health System Database

Exclusion criteria involved studies that attempted to predict non-suicidal self injury (NNSI) or suicidal ideation that did not lead to a suicide attempt or taking one's life. Some studies provided prediction ratings for suicidal ideation alongside suicide risk behaviors and were included, although the ideation data in these studies were not used. Furthermore, papers that were suggested to have the potential for overestimation of predictive accuracy through the use of certain algorithms were excluded.

### Data extraction

A spreadsheet was determined *a priori* to extract data from the studies. Information extracted included author details, the title of the paper, year of publication, country of origin, sample size, and demographics. The primary method of assessing accuracy was through the use of the area under the curve (AUC), sensitivity, and specificity scores. Other outcome scores included positive predictive value (PPV), negative predictive value (NPV), and accuracy. Confusion matrix data was extracted from the majority of studies, and a cut-point was calculated from sensitivity and specificity for continuous data were not originally included in papers. Confusion matrix data are outlined in Supplementary material.

### Statistical analysis

Meta-analysis was conducted using R version 1.4 (56). To begin, extracted data were classified into 2 x 2 “confusion matrix” tables from each of the eligible 31 studies. The tables defined the presence or absence of a condition (suicidal or not suicidal) related to four outcomes, namely, true positives (TP), false positives (FP), false negatives (FN), and true negatives (TN). Figures were extracted from studies and used original authors cut-points. In a minority of cases, cut-points were calculated by multiplying the sensitivity score × total suicidal population and rounding to the nearest whole number to obtain TP and FN scores. Multiplication of the specificity figure × the total non-suicidal population provided the FP and TN figures (57). Confusion matrix data was assessed for imbalanced data (which can inflate accuracy scores due to imbalances between the number of cases and controls) and calculated (TPR = TN / TN + FN) + (TNR = TN/TN + FP)/2 to obtain balanced accuracy scores.

The R package “*mada”* was used for calculating forest plots, meta-analysis, and meta-regression. The package was selected for all analyses consistent with published protocols (28, 57) regarding the “gold standard” use of bivariate analysis in diagnostic accuracy studies. Bivariate analysis is defined by the inclusion of both the sensitivity and the specificity figures as an approach to assessing an overall AUC outcome. These two statistics relate to each other through a cut-point value, such that as sensitivity increases, specificity decreases. The final model was implemented in *mada*'s Reitsma function (57).

The overall data analysis strategy was to combine sensitivity and specificity and run a bivariate meta-analysis to attain total AUC accuracy scores overall and for each data source. This was done through the calculation of a summary receiver operating characteristic (SROC) curve. This curve is the graphical representation of a meta-analysis for a diagnostic test (58).

An assessment for heterogeneity was then undertaken (28). Of note, the standard psychometrics of heterogeneity (Cochranes *Q* and *I*^2^) are not appropriate when using a bivariate approach. Consequently, the SROC curve was used to assess heterogeneity. According to Shim, Kim and Lee (28) there are four methods of assessing for heterogeneity when using a bivariate approach: the asymmetry of the SROC curve; a wide degree of scattering of individual studies in the SROC curve; if between-study variation is greater than within-study variation as observable in the forest plots; and if the correlation coefficient (*r*) of sensitivity and specificity is larger than 0, indicating a relationship. The correlation coefficient is always a negative number in a bivariate approach as the two figures are balanced against each other—as one increases, the other decreases (28).

The final step in the data analysis plan was to investigate the potential sources of heterogeneity (if such exist) through bivariate random-effects meta-regression. This analysis investigates the impact of moderators on outcomes. Study-level characteristics (such as data source used to train the algorithms) and participant-level characteristics (including sex—the percentage of female participants, the outcome of the study—attempt suicide/risk behavior or taking one's life, and the setting in which data was obtained—inpatient/outpatient or outpatient only) were used as potential explanatory moderators, consistent with the method outlined by Debray et al. (59).

## Results

**Figure 1:**
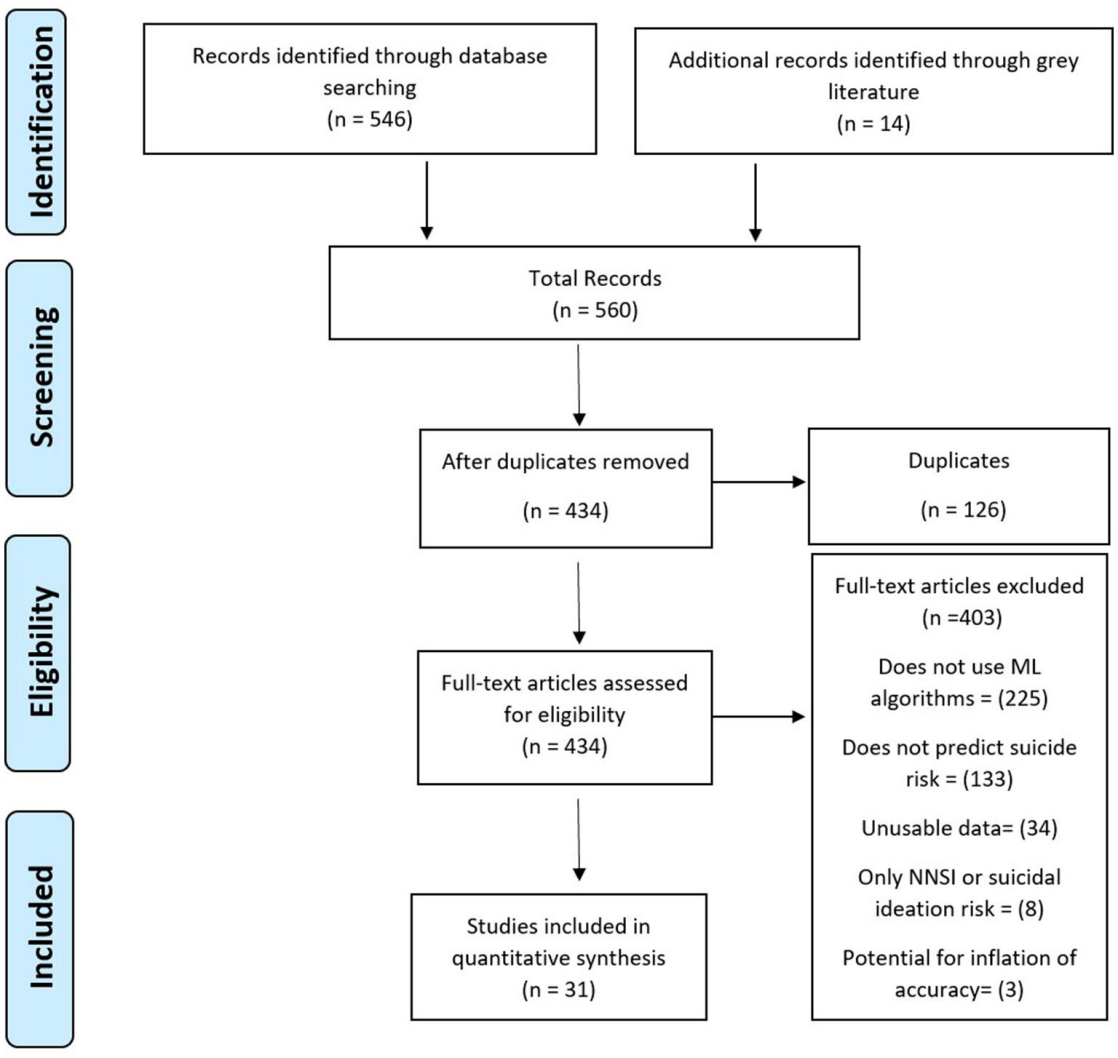
PRISMA diagram of literature search.

The initial search yielded 560 studies. Figure 1 shows the literature search results. After duplicates were removed, 434 studies underwent full-text screening by the first author. In total, 403 articles were excluded: 225 that did not use algorithms, 133 that did not predict suicide risk, 34 that did not contain adequate data, eight that focused only on self-injury or suicidal ideation, and three that were suggested to be overestimated given the type of algorithm and validation methods used.

### Study participants and populations

All 31 studies were modeling studies and had a total sample size of 11,163,953 with a mean of 360, 128 and a range of 73–2,960,929. The majority were conducted in the USA (13), followed by Korea (5), Canada (2), Chile (2), France (2), the Netherlands (2), with China, Iran, Sweden, Australia, and Spain producing one study each.

Twenty-three studies (*n* = 9,566,166) included gender demographics, with a mean of 58.41% participants being female. Twenty-one studies included only adult samples, seven included both adolescent and adult samples, and three studies exclusively sampled adolescents. The study participants were drawn from 16 outpatient services and 15 combined inpatient/outpatient service locations.

There were 31 studies identified as meeting the selection criteria that were included in the meta-analysis. To train the algorithms, 15 of the studies used structured data and 16 studies used unstructured data as outlined in Table 2. There were 25 studies that predicted suicidal behavior as an outcome, four studies focused on the prediction of suicide death, and two on suicide attempt/death combined.

**Table 2:** Study characteristics.

**Study**	**Authors**	**Outcome**	**Total *N***	**Balanced accuracy**	**Population female**	**Mean age**	**Sample age range**	**Service location**	**Country**	**Algorithm**	**Validation method**
**Structured data**											
1.	Barros et al., (29)	SA/R	707	0.79	564	39.7	Adult	Inpatient/outpatient	Chile	Support vector machine	K-fold cross validation
2.	Barros et al., (31)	SA/R	650	0.79	517	39.77	Adult	Inpatient/outpatient	Chile	Support vector machine	K-fold cross validation
3.	Burke et al., (33)	SA/R	25,326	0.87	15,182	16.43	14 to 24	Outpatient	USA	Decision tree Random forest Ridge regression	Repeated cross validation
4.	Delgado-Gomez et al., (35)	SA/R	902	0.87	470	38.5	Adult	Inpatient/outpatient	Spain	Decision tree	Cross validation
5.	Hill et al., (37)	SA/R	4,834	0.65	2,528	16.15	Adolescent	Outpatient	USA	Decision tree	10-fold cross validation
6.	Horvarth., (39)	SA/R	353	0.87	207	^*^	Adult	Inpatient	Australia	XGBoost	*
7.	Jung et al., (41)	SA/R	59,984	0.78	29,600	15	Adolescent	Outpatient	Korea	XGBoost	Pairing Cross Validation
8.	Kim et al., (43)	SA/R	7,824	0.78	4,139	19.57	18-25	Outpatient	Korea	Random forest K nearest neighbor	*
9.	Morales et al., (45)	SA/R	707	0.68	564	39.7	14-83	Outpatient	France	Decision tree	Cross validation
10.	Naghavi et al., (47)	SA/R	573	0.89	419	24.45	Adult	Outpatient	Iran	Stacked decision tree	K-fold cross validation
11.	Oh et al., (49)	SA/R	573	0.91	306	35.6	Adult	Outpatient	Korea	Neural network	Cross validation
12.	Passos et al., (9)	SA/R	144	0.71	91	36.4	Adult	Outpatient	USA	Relevance vector machine	Cross validation
13.	Rozek et al., (51)	SA/R	152	0.66	19	27.4	19–44	Outpatient	USA	Mondobrain	*
14.	Ryu et al., (53)	SA/R	5,773	0.92	^*^	^*^	Adolescent	Outpatient	Korea	Random forest	10-fold cross validation
15.	Shen et al., (54)	SA/R	4,882	0.78	4,345	18.77	>15	Outpatient	China	Random forest	5-fold cross validation
**Unstructured data**											
16.	Barak- Corren et al., (30)	SA/R	1,728,549	0.71	1,005,992	^*^	10 to 89	Inpatient/outpatient	USA	Naïve bayes	Cross validation
17.	Barak-Corren et al., (32)	SA/R	3,714,105	0.66	2,130,454	^*^	Adult	Outpatient	USA	Naïve bayes	Cross validation
18.	Carson et al., (34)	SA/R	73	0.80	45	15.94	Adolescent	Inpatient	USA	Natural language processing	5-fold cross validation
19.	Chen et al., (36)	SA/R and SD	541,300	0.73	305,299	27.3	Adult	Inpatient/outpatient	Sweden	Elastic net Random forest Gradient boosting Neural network	10-fold cross validation
20.	Cho et al., (38)	SA/R	372,813	0.79	179,122	64.23	Adult	Outpatient	Korea	Elastic Net Random forest Gradient boosting Neural network	Cross validation
21.	Kessler et al., (40)	SD	975,057	0.67	^*^	^*^	Adult	Outpatient	USA	Decision tree	5-fold cross validation
22.	Kessler et al., (42)	SD	391,018	0.59	^*^	^*^	Adult	Inpatient	USA	Naïve bayes Random forest	Cross validation
23.	Metzger et al., (44)	SA/R	20,254	0.97	^*^	^*^	>15	Inpatient/outpatient	France	Naïve bayes Random forest	Cross validation
24.	Sanderson et al., (46)	SD	39,028	0.76	^*^	^*^	Adult	Inpatient/outpatient	Canada	Hidden layer neural network	Cross validation
25.	Sanderson et al., (48)	SD	30,694	0.79	^*^	^*^	Adult	Inpatient/outpatient	Canada	Logistic Regression	10-fold cross validation
26.	Simon et al., (23)	SA/R and SD	2,960,929	0.76	1,835,776	^*^	13 to 65	Outpatient	USA	Logistic regression	10-fold cross validation
27.	Su et al., (50)	SA/R	41,721	0.70	20,753	^*^	Adolescent	Inpatient/outpatient	USA	Logistic regression	10-Fold Cross Validation
28.	Tsui et al., (52)	SA/R	45,238	0.85	27,266	^*^	10–75	Inpatient/outpatient	USA	Extreme gradient boosting	5-fold cross validation
29.	Van Mens et al., (4)	SA/R	725	0.70	^*^	^*^	Adult	Outpatient	Netherlands	Random forest	Cross validation
30.	Van Mens et al., (4)	SA/R	53,827	0.68	120,549	49	Adult	Outpatient	Netherlands	Random forest	5-fold cross validation
31.	Zheng et al., (55)	SA/R	118,095	0.60	^*^	^*^	Adult	Inpatient/outpatient	USA	Deep neural network	Cross validation

* Information not reported in original papers.

SA/R, Suicide Attempt/Risk; SD, Suicide Death.

### Data source moderators

The structured data studies (15 studies) used standardized risk psychometrics to inform algorithms (*n* = 108,182) to predict suicide attempt/risk behavior. The unstructured data studies (*n* = 11,055,771) used EHR and databases to predict suicide behaviors, including suicide attempt/risk (10 studies) suicide attempt/death combined (two studies), and suicide death (four studies). They included all medical records and administrative information held in databases at hospitals, general practitioners, corrective services, the armed forces, or any other institution where medical notes might be stored. Some of the included studies attempted to account for moderators such as age and gender within their designs, however, this did not affect the current review as only the total model outcomes were considered for meta-analysis and meta-regression.

### Meta-analysis of suicide prediction model accuracy

**Figure 2:**
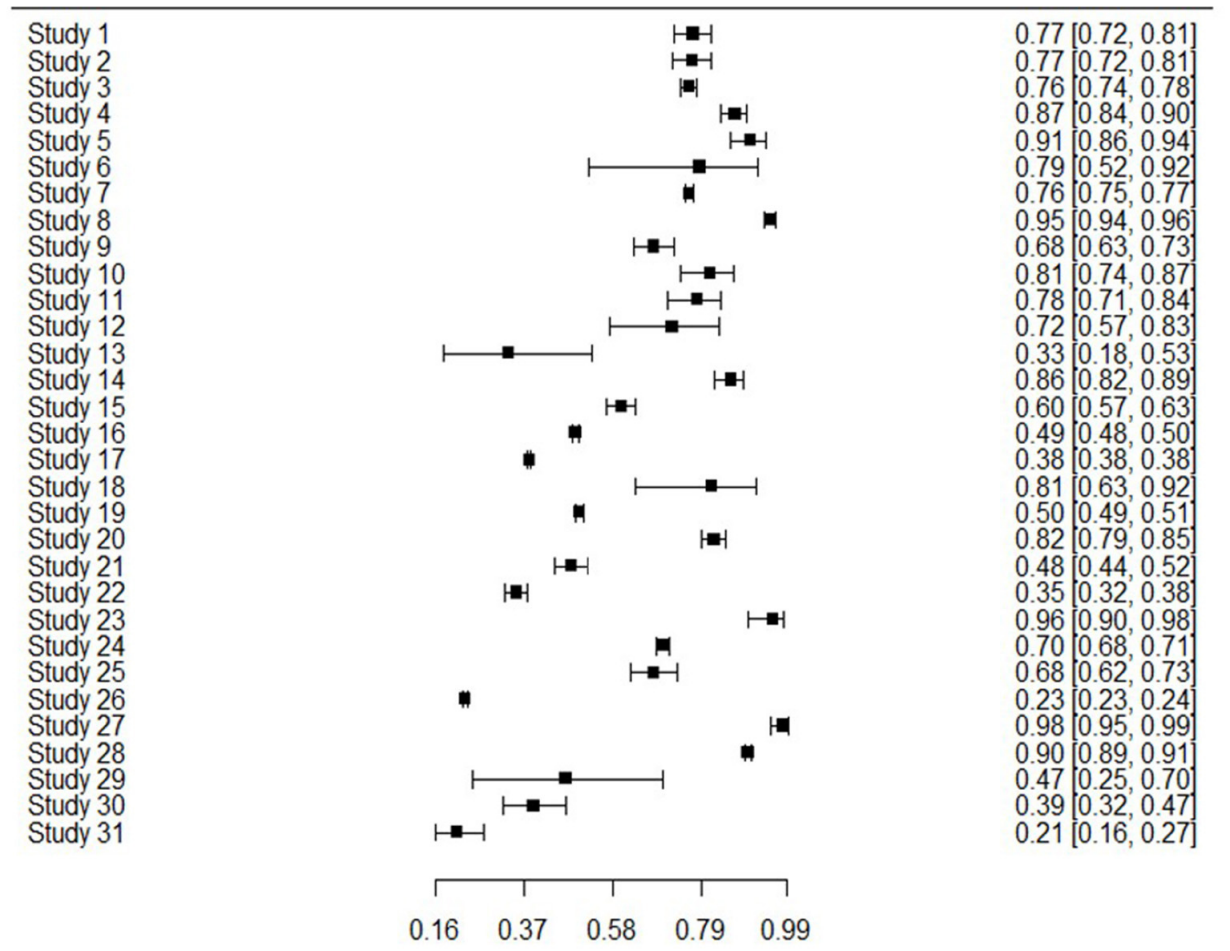
Forest plot of sensitivity and CI for 31 studies.

**Figure 3:**
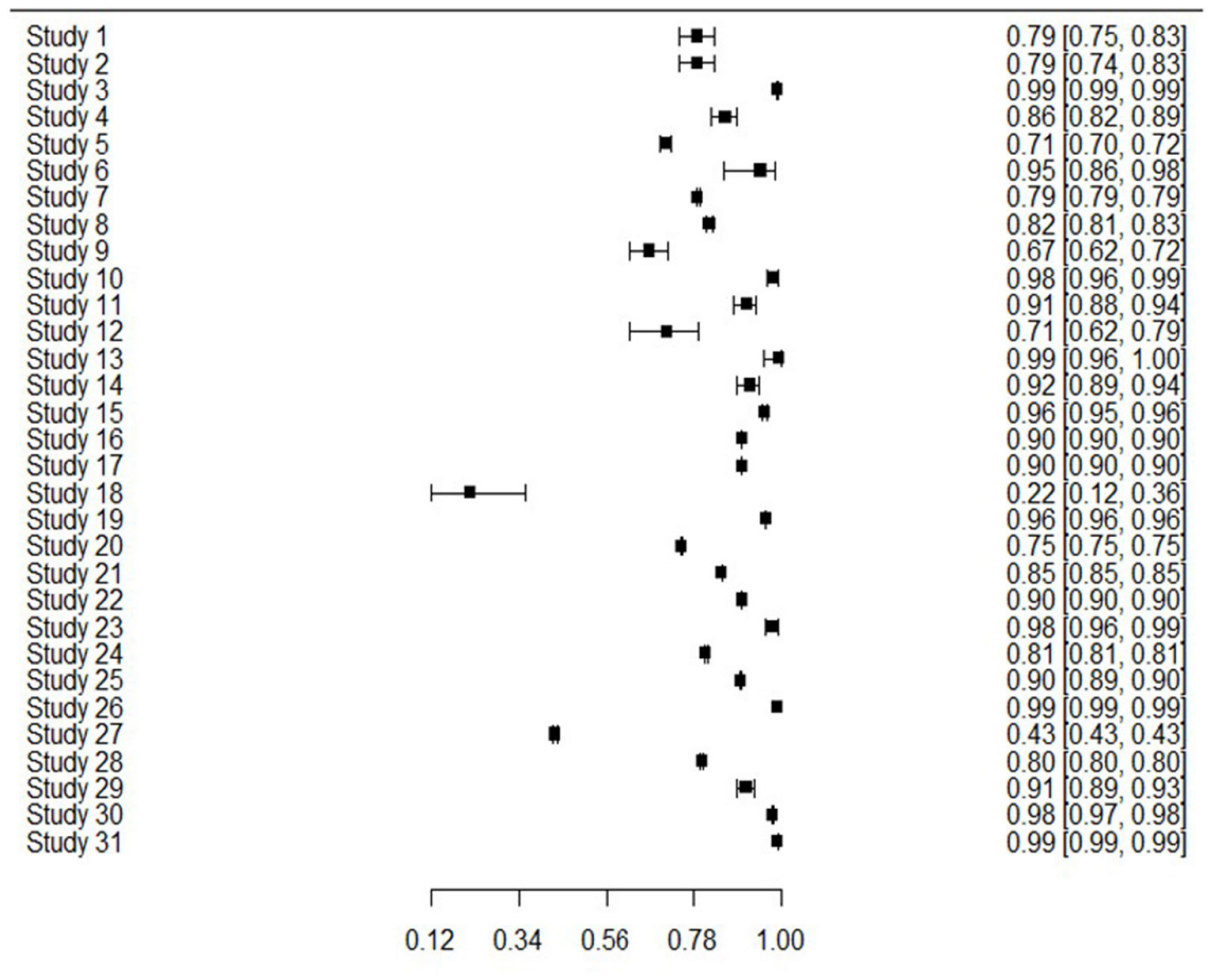
Forest plot of specificity and CI for all 31 studies.

The 31 studies were able to be used in the meta-analysis as informed by the combination of sensitivity and specificity to produce bivariate outcomes and an AUC. Sensitivity or true positive rate, is the ability to identify those with the outcome of interest, whereas specificity or true negative rate, is the ability to correctly identify those without the outcome of interest. Figure 2 presents sensitivity for all studies, showing that generally sensitivity varied widely from 0.21 to 0.94, but was mostly in the 0.70 s. Specificity values are shown in Figure 3, revealing a range from 0.22 to 0.99, but most often high in the 0.90 s, with low scores being few. It is important to note that for events with low base rates such as suicidal behaviors, specificity is not always a useful indicator of accuracy. However, such is an inherent limitation in research of rare events.

### Meta-analyses and SROC curve

**Figure 4:**
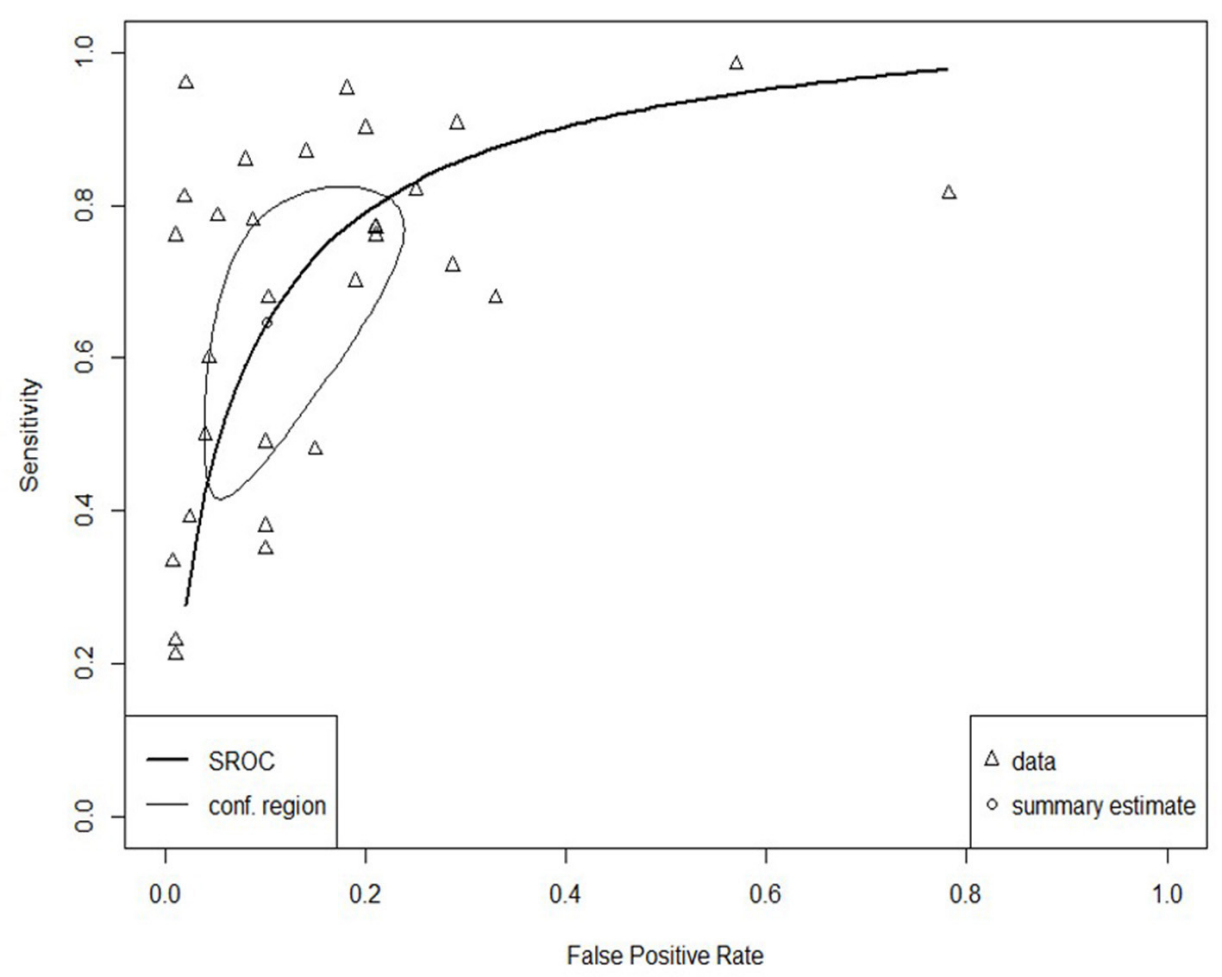
SROC curve for all studies (*n* = 31).

**Figure 5:**
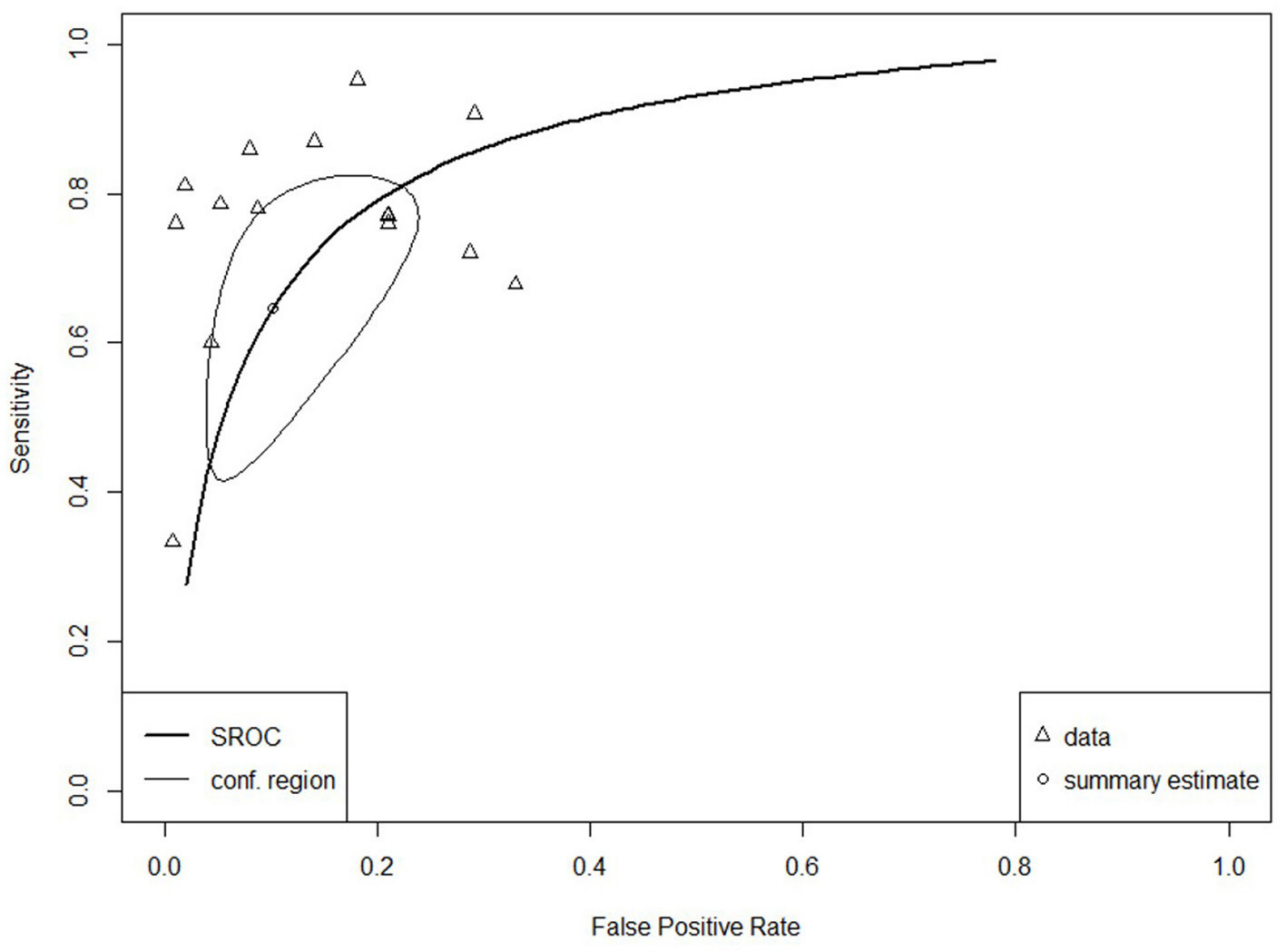
SROC curve for structured data (*n* = 15).

**Figure 6:**
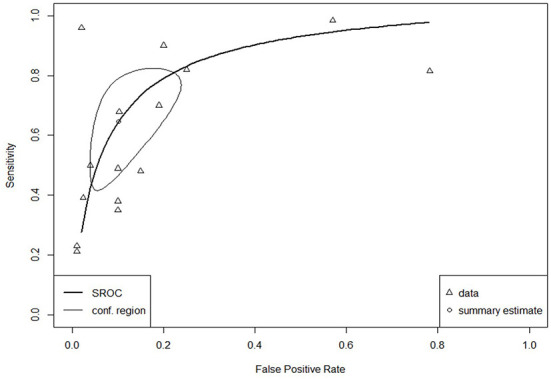
SROC curve for unstructured data (*n* = 16).

The SROC curve for the total studies, showing the prediction area with 95% confidence of the true sensitivity and specificity, is provided in Figure 4. Meta-analysis results revealed an overall AUC = 0.860 for the 31 studies, which is in the “good” range. Each data type also underwent meta-analysis to investigate potential accuracy differences between the two groups. Structured data as outlined in Figure 5 (AUC = 0.873) and unstructured data as outlined in Figure 6 (AUC = 0.866) both showed outcomes in the “good” range and were quite similar in their accuracy outcomes.

### Heterogeneity

The SROC curve and distributions for the studies were assessed for heterogeneity according to the aforementioned four components (28). Substantial between-study variance was evident through the asymmetry of the SROC curve, the wide scattering of the studies, the visually larger disparity in the between-study variation, and a moderately strong, negative correlation coefficient of sensitivity and specificity of −0.64 (well above the required score of 0).

### Bivariate random-effects meta-regression

Meta-regression was undertaken on available moderators (structured and unstructured data, study outcome, percentage females, participant age, and service location) to attempt to account for some of the heterogeneity between the 31 studies. There were no significant results as outlined in Table 3, as *z* scores were >0.05 for all moderators.

**Table 3:** Bivariate meta-regression moderators.

**Moderators**	**Included studies**		**Estimate** **Standard** **Error**	**95%** **Confidence** **Interval**	***z*-score**	**Pr (>|z|)**
Data source	Structured data= 15 Unstructured data= 16	Sens	−0.579	−1.429 lb 0.271 ub	−1.334	0.182
		Tfpr	0.100	−0.963 lb 1.163 ub	0.184	0.854
Outcome	Attempt suicide/risk *=* 25 Suicide death = 6	Sens	−1.139	−2.139 lb −0.138 ub	−2.231	0.226
		Tfpr	−0.436	−1.667 lb 0.399 ub	−0.657	0.511
Percentage female	Total = 23	Sens	−0.002	−1.736 lb 0.0865 ub	−0.091	0.927
		Tfpr	0.010	−0.035 lb 0.054 ub	0.425	−0.671
Participants mean age	Total = 17	Sens	0.349	−0.050 lb 0.037 ub	−0.307	0.759
		Tfpr	0.289	−0.043 lb 0.061 ub	0.334	0.738
Service location	Inpatient/outpatient =14 Outpatient = 17	Sens	−0.511	−1.384 lb 0.361 ub	−1.148	0.251
		Tfpr	−0.634	−1.667 lb 0.399 ub	−1.202	0.229

Sens, sensitivity; Tfpr, true false positive rate; lb, lower bound; up, upper bound.

### Potential for bias

Risk of bias (ROB) was assessed by two authors (DH, CW) according to the prediction model study risk of bias assessment tool (PROBAST) developed by Wolff et al. (60). The PROBAST is designed to assess both the ROB and any concerns regarding the applicability of studies that either develop, validate, or update a previous prediction model. Studies are rated according to four domains for both ROB and applicability: participants, predictors, outcomes, and analysis (61). Overall, there was a high ROB observed in the majority of studies. The reasons for this were similar across studies. Most studies had knowledge of the outcome prior to the study commencing given that participants had either taken their life or attempted to do so, which increased the ROB in almost all cases. Consequently, in these types of retrospective studies, ROB is inevitably elevated.

Of the 16 unstructured data studies, 11 were high ROB, one was low ROB, and four were unclear. Regarding the 15 structured data studies, nine were considered high ROB, and six were low ROB as outlined in Table 4.

**Table 4:** PROBAST risk of bias analysis.

	**Risk of bias**	**Applicability**
Barros et al., (29)	**−**	**+**
Barros et al., (31)	**−**	**+**
Burke et al., (33)	**+**	**+**
Delgado-Gomez et al., (35)	**−**	**+**
Hill et al., (37)	**+**	**+**
Horvarth., (39)	**+**	**+**
Jung et al., (41)	**−**	**+**
Kim et al., (43)	**−**	**+**
Morales et al., (45)	**−**	**?**
Naghavi et al., (47)	**−**	**?**
Oh et al., (49)	**+**	**+**
Passos et al., (9)	**+**	**+**
Rozek et al., (51)	**+**	**+**
Ryu et al., (53)	**+**	**+**
Shen et al., (54)	**−**	**?**
Barak-Corren et al., (30)	**−**	**+**
Barak-Corren et al., (32)	**−**	**+**
Carson et al., (34)	**?**	**+**
Chen et al., (36)	**?**	**+**
Cho et al., (38)	**+**	**+**
Kessler et al., (40)	**−**	**+**
Kessler et al., (42)	**?**	**+**
Metzger et al., (44)	**−**	**?**
Sanderson et al., (46)	**−**	**+**
Sanderson et al., (48)	**−**	**+**
Simon et al., (23)	**−**	**+**
Su et al., (50)	**−**	**+**
Tsui et al., (52)	**−**	**+**
Van Mens et al., (4)	**−**	**+**
Van Mens et al., (4)	**?**	**+**
Walsh et al., (10)	**−**	**+**
Zheng et al., (55)	**−**	**+**

### Publication bias

It is not possible to assess for publication bias in bivariate meta-analysis with accuracy at this stage. According to the Cochrane Handbook for Systematic Review of Diagnostic Test Accuracy (62), there are precision concerns with attempting traditional publication bias analysis on diagnostic test accuracy studies. Previously, Deeks (63) highlighted that applying funnel plots or using other traditional statistical tests was likely to result in publication errors being indicated incorrectly through type 1 errors. The alternative method proposed was to test the association between the diagnostic odds ratio (DOR) and the sample size. However, when there is heterogeneity present, the usefulness of such a method is rendered negligible due to minimized power (63). Given the current study has revealed a high amount of heterogeneity and did not include calculations of DOR as it was a bivariate approach, as assessment of publication bias was not able to be undertaken.

## Discussion

This systematic review addresses a gap in the field of ML in suicide risk prediction by considering the predictive accuracy overall and through a comparison of two types of data sources using meta-analytic investigation. Most studies were on unstructured data and almost as many used structured data. As suicide attempt behavior and death are rare occurrences statistically, studies adopted a retrospective approach, in that the outcome was already known prior to the development of the algorithms, increasing the ROB in most studies.

Meta-analysis of the 31 studies showed a “good” AUC rating overall, better than the unstructured clinical judgment of suicide risk, which is suggested to be no better than chance (AUC = 0.5) (3). The current meta-analytic result also suggested improving upon reported structured psychometric accuracy figures (AUC = 0.70–0.80) (14). Of note, a meta-analysis suggested similar accuracy levels in the “good” range for both structured and unstructured data sources. According to the current review, using either of the data sources in algorithms produces similar or better accuracy in the detection of suicide risk behaviors than clinical judgment and structured risk assessment undertaken by a clinician. This is an important finding for clinical practice, given that with reliance on only self-report, fewer than expected “high risk” clients complete suicide, while a number of “low risk” clients do go on to complete suicide (12, 13). Therefore, the combination of clinical judgment, psychometric use and development of algorithms may increase detection of suicide in certain cases. Nevertheless, further research and refinement of algorithms validated with different populations and in various settings over time are important before any clinical implementation can be attempted.

Considerable heterogeneity was detected through examination of the SROC curve of the 31 studies, as well as through interpretation of the correlation coefficient. The moderators that were analyzed—including structured data, unstructured data, percentage females, mean age, service location, and study outcome—did not show any significant effects, suggesting that none of these accounted for the heterogeneity in predictive accuracy. It is likely that the sources of variance between the studies were due to variability in individual level characteristics, study design, or issues related to publication (including the risk of bias) (59). Given the majority of the studies used a retrospective approach, heterogeneity may have been more likely because of sampling bias and/or impaired study design as the population and designs were restricted by the already known outcome. Investigation of sources of difference between studies is important as it may highlight significant areas of convergence and divergence in suicide risk prediction and presents as a limitation in interpreting the results.

Consideration of the two types of data sources provided some interesting findings. Compared to the unstructured data studies, which tended to be entire electronic health records often detailed by an external person, structured data studies were specific and subjective data sources, given their basis in individual self-report or expression. Superficially, similar accuracy scores between structured data and unstructured data may suggest equivalent predictive ability, although it is noted that the data sources do not have comparable input data. The type of input data required for good predictive outcomes in structured data is generally modest and involves one or more targeted and specific psychometric instruments. To achieve similar predictive outcomes for unstructured data, algorithms were provided with substantially more unorganized data, from larger populations, to recognize patterns and detect suicide risk. Overall, this suggests that the use of structured, specific data may be sufficiently accurate for suicide risk detection if such is able to be collected by a service over time. However, unstructured data use may be a faster method of highlighting risk, if it can be generalized to a certain population.

The potential for translation of this and similar research into clinical practice aid in highlighting implementation considerations. While structured data showed just as good accuracy as unstructured, such specific information is not always available when attempting to predict and manage suicide risk behaviors. Further, consistent with prior work on suicide risk factors, history of suicide risk and self-harm behaviors were not strong predictors in the majority of the studies, and having an objective way of assessing risk is therefore important (2). Presentation to hospital emergency, help-seekers of crisis support services, intake into the prison system, or initial engagement with school counseling or community mental health services, would require time to build specific structured data related to a population, and only if the organization kept records (not often the case for crisis support). However, if unstructured data could be used to estimate suicide risk behavior at a population level through algorithms, intervention and further assessment may be prompted, regardless of lack of information or help-negation of clients (5). In such situations, clinicians may not always need to rely entirely on self-report methods for risk assessments to achieve accurate detection levels of suicide risk. For instance, “at-risk” clients highlighted through ML may then be flagged for further assessment or specific support, both areas for future consideration. In this way, ML may complement existing risk assessment methods by allowing clinicians another point of data when considering suicide risk. The use of algorithms is considered by the authors to be potentially complementary to clinical practice, rather than a means of replacement of clinical judgment, therapeutic trust and rapport, or individual risk instruments.

The results of the current review must be considered in light of its limitations. A limitation of note was the lack of standardization of risk variables/input data/populations across studies. It seems likely that studies, especially unstructured data compared to structured data, could be measuring the same variables, using different semantic labels, or by asking direct questions about suicide in clinical notes, rather than using psychometric instruments. Regardless, the study highlighted that both structured and unstructured data produce “good” accuracy levels in predicting suicide risk.

Another limitation of the current review was that the commonly used retrospective method led to high ROB results for the majority of studies. The ROB exists in these studies given that outcomes must be known in advance (suicidal risk behavior or death) to allow accurate predictions to be validated. Any study design with a known outcome is inherently biased toward that result. Prospective studies of suicide are, however, very challenging given the fortunate rarity of the events statistically. The confines of statistical analysis when studying events with such low base rates are also highlighted as a limitation.

Other limits of the research field identified by this review are that studies were highly context-specific, potentially minimizing their generalizability and use in clinical practice at this stage. The results must be viewed cautiously given the large heterogeneity between the studies, which could not be accounted for through meta-regression. Further, suicide risk factors and content of data in both structured and unstructured data types varied across cultures and nations, which necessitates specific prediction models to be developed for each region to which they are attempting to apply (48). The use of algorithms in clinical practice is likely some time away given each context will require validation to apply to specific populations to ensure accuracy.

Overall, taking into account limitations, this review presents promising findings for the accuracy of ML algorithms with both structured and unstructured data sources in suicide risk prediction, although other obstructions to implementation exist. Several clinical factors are major barriers, including a range of privacy, consent, and practical considerations for clients (64), as well as implications for legal responsibility, safety, and algorithm accuracy on an individual level (15). In addition, clinician attitudes toward ML use and the transition of ethical responsibilities and principles from in-person clinical interactions, to those that are technologically assisted have been raised (65). Given the ethical treatment of clients is paramount in all clinical fields, such is vital to explore. It is therefore of importance that future research focuses on potential barriers to ML use in clinical environments including the practicality of use and general uptake motivation of clients and clinicians.

In conclusion, the review revealed good accuracy scores for ML algorithms, equal to, or higher than stand-alone suicide risk psychometrics and/or clinician judgment. Both structured and unstructured data sources showed similar accuracy outcomes, despite different levels of data organization and specificity regarding the outcome of suicide risk prediction. Given suicide continues to be a leading cause of preventable death globally and there has been little improvement in detecting suicide risk over time, investigation of innovative, technology-based methods is important for evolving clinical practice.

## Data availability statement

The original contributions presented in the study are included in the article/Supplementary material, further inquiries can be directed to the corresponding author.

## Author contributions

DH designed the review, undertook the literature searches and screening, and wrote the protocol, as well as the various drafts of the manuscript. DR supervised the development, conceptualization, and preparation of the review. DH completed the statistical analysis under the supervision of DJH. DH and CW completed the risk of bias assessments for all studies. All authors contributed to the editing, final draft, and have approved the final manuscript.

## Conflict of interest

The authors declare that the research was conducted in the absence of any commercial or financial relationships that could be construed as a potential conflict of interest.

## Publisher's note

All claims expressed in this article are solely those of the authors and do not necessarily represent those of their affiliated organizations, or those of the publisher, the editors and the reviewers. Any product that may be evaluated in this article, or claim that may be made by its manufacturer, is not guaranteed or endorsed by the publisher.

## Supplementary Material

SUPPLEMENTARY TABLE 1 Confusion matrix data.
